# Maternal High-Protein and Low-Protein Diets Perturb Hypothalamus and Liver Transcriptome and Metabolic Homeostasis in Adult Mouse Offspring

**DOI:** 10.3389/fgene.2018.00642

**Published:** 2018-12-11

**Authors:** Lisa J. Martin, Qingying Meng, Montgomery Blencowe, Sandrine Lagarrigue, Sheila Xiao, Calvin Pan, Julian Wier, William C. Temple, Sherin U. Devaskar, Aldons J. Lusis, Xia Yang

**Affiliations:** ^1^Department of Medicine, Division of Cardiology, David Geffen School of Medicine, University of California, Los Angeles, Los Angeles, CA, United States; ^2^Department of Integrative Biology and Physiology, University of California, Los Angeles, Los Angeles, CA, United States; ^3^INRA, Agrocampus Ouest, UMR1348 PEGASE, Rennes, France; ^4^Department of Pediatrics, David Geffen School of Medicine, University of California, Los Angeles, Los Angeles, CA, United States; ^5^Department of Human Genetics, David Geffen School of Medicine, University of California, Los Angeles, Los Angeles, CA, United States; ^6^Department of Microbiology, Immunology and Molecular Genetics, David Geffen School of Medicine, University of California, Los Angeles, Los Angeles, CA, United States

**Keywords:** maternal diet, high protein, low protein, gene expression, metabolic dysfunction, glucose intolerance, hypothalamus, liver

## Abstract

Early life nutritional imbalances are risk factors for metabolic dysfunctions in adulthood, but the long term effects of perinatal exposure to high versus low protein diets are not completely understood. We exposed C57BL/6J offspring to a high protein/low carbohydrate (HP/LC) or low protein/high carbohydrate (LP/HC) diet during gestation and lactation, and measured metabolic phenotypes between birth and 10 months of age in male offspring. Perinatal HP/LC and LP/HC exposures resulted in a decreased ability to clear glucose in the offspring, with reduced baseline insulin and glucose concentrations in the LP/HC group and a reduced insulin response post-glucose challenge in the HP/LC group. The LP/HC diet group also showed reduced birth and weanling weights, whereas the HP/LC offspring displayed increased weanling weight with increased adiposity beyond 5 months of age. Gene expression profiling of hypothalamus and liver revealed alterations in diverse molecular pathways by both diets. Specifically, hypothalamic transcriptome and pathway analyses demonstrated perturbations of MAPK and hedgehog signaling, processes associated with neural restructuring and transmission, and phosphate metabolism by perinatal protein imbalances. Liver transcriptomics revealed changes in purine and phosphate metabolism, hedgehog signaling, and circadian rhythm pathways. Our results indicate maternal protein imbalances perturbing molecular pathways in central and peripheral metabolic tissues, thereby predisposing the male offspring to metabolic dysfunctions.

## Introduction

Metabolic syndrome and type 2 diabetes mellitus (T2DM) have reached epidemic proportions worldwide (Saklayen, 2018). A major risk factor for the development of T2DM is the quantity and quality of intra-uterine nutrient exposure (Gluckman et al., 2008). Studies have shown that the maternal diet can modify the development of regulatory systems *in utero* and postnatally. Individual studies of high and low protein maternal diets have shown their influence on body weight, metabolic phenotypes and programming of food intake in offspring (Oken and Gillman, 2003; Frias and Grove, 2012). Our previous mouse study on intrauterine growth restriction (IUGR) induced by a low protein maternal diet demonstrated sex-specific phenotypic differences in offspring at 9 months of age (Bhasin et al., 2009). While the male offspring displayed glucose intolerance and increased adiposity, the female offspring were glucose tolerant, without catch-up growth or adiposity. However, the mechanisms underlying these metabolic changes are yet to be elucidated. In addition, previous studies have not systematically compared the effect of low protein/high carbohydrate (LP/HC) versus high protein/low carbohydrate (HP/LC) maternal diets on modulating tissue-specific gene expression profiles in key metabolic tissues and the associated pathological metabolic variations in offspring. As gene expression differences involved in metabolically relevant pathways in the hypothalamus (the control center of energy balance and metabolism) and liver (critical for lipid and glucose homeostasis) may underlie the differing metabolic phenotypes in adulthood, we aim to investigate how early-life nutritional imbalances contribute to metabolic syndrome outcomes in adulthood.

In the current study, we tested the hypothesis that intrauterine HP/LC and LP/HC diets would subsequently affect adult metabolic phenotypes and long-term gene expression levels in key metabolic sites. Using HP/LC or LP/HC dietary interventions during gestation and lactation, we followed male offspring until 10 months of age and assessed body weight, adiposity, insulin production, glucose clearance, and global gene expression changes in the hypothalamus and liver. We discovered that mice expressed differential programming by perinatal exposure to protein imbalances, and these changes persist into adulthood. Importantly, we found that gene expression changes brought about by perinatal nutritional perturbations revealed enrichment of genes involved in diverse pathways including those important for systemic energy homeostasis.

## Materials and Methods

### Study Approval

This study was approved by the UCLA Animal Research Committee and was performed in accordance with the National Institutes of Health guidelines for the use of experimental animals. C57BL/6J mice were purchased from the Jackson Laboratory (Bar Harbor, ME, United States).

### Diets

The LP/HC diet (D02041002, Research Diets Inc., New Brunswick, NJ, United States) contains 9% protein, 4.4% fat, and 77% carbohydrates. The HP/LC diet (D02041001, Research Diets Inc., New Brunswick, NJ, United States) has 23% protein, 4.4% fat and 64% carbohydrate by weight. Standard chow diet (TD 7013, Harlan Teklad, Placentia, CA, United States), containing 18% protein, 6% fat, and 45% carbohydrate by weight, was used as the control diet. The diets were matched for total caloric content, making them isocaloric.

### Maternal Diet Exposure Studies

As most first litters did not survive, all the offspring were from second litters from dams 10 to 12 weeks old. C57BL/6 females were mated overnight with males between 10 and 16 weeks of age. Gestational day 0 (GD0) was determined by the detection of a vaginal plug in the morning. On GD8, pregnant females were placed on a LP/HC, HP/LC, or chow diet. Pups were weaned at 28 days of age into cages of four animals per cage, separated by sex and maternal dietary environment. A total of *n* = 22, *n* = 27, and *n* = 20 male offspring from the LP/HC, HP/LC, or chow diet group, respectively, were examined.

### Body Weight and Body Composition

Body weight of individual mice was measured on a scale, starting day 2 (birth weight) and thereafter monthly until sacrifice. Body composition was measured monthly until sacrifice by a rodent Nuclear Magnetic Resonance (NMR) scanner (Bruker Biospin, Billerica, MA, United States) that was standardized to an internal control provided by the manufacturer. Adiposity was determined as adipose tissue mass per unit (g) body weight.

### Glucose Tolerance Tests and Insulin Measurement

Glucose tolerance tests were performed as previously described (Ganguly and Devaskar, 2008) in 9-month-old male offspring. After an overnight fast, glucose was measured at 9AM in blood collected from saphenous venous puncture, after which 2 mg/g body weight of glucose was administered intraperitoneally. Blood glucose was measured at half hour intervals for 2 h. The One Touch Ultra (LifeScan) glucometer was used to measure whole blood glucose concentrations (Ganguly and Devaskar, 2008). Insulin was measured with an ELISA kit (ALPCO, Salem, NH #80-INSMSU-E10) using 5 μL of plasma separated from blood collected at baseline and at 30 min post-glucose challenge.

### Phenotypic Data Analysis

All figures demonstrate data displayed as means ± SEM, with the exception of insulin concentrations that were expressed as a ratio of 30 min/baseline values in Figure 2, indicating medians. The two-way ANOVA model was employed for growth curves and glucose tolerance test curves, to simultaneously compare the effect of diets (treatment) and time points within each group. GraphPad Prism version 7.0 software was used for graphing and for statistical analysis of phenotypic data.

### Microarray Analysis

Hypothalamus and liver were dissected between 10 AM and 12 PM from male mice at 10 months of age, placed immediately into liquid nitrogen, and stored at -80°C until RNA was extracted with the RNAeasy kit (Qiagen). A total of 40 males from three different *in utero* exposure groups were analyzed for gene expression changes in hypothalamus and liver (15, 15, and 10 for LP/HC, HP/LC, and Control, respectively) using the Affymetrix Mouse 430 2.0 microarray chip. The chip contained 45,000 probes representing 35,000 genes. *P*-values for differential gene expression were determined using the Student’s *t*-test for pairwise comparisons, each treatment compared with control, followed by multiple testing correction using the *q*-value approach (Storey, 2003). For pathway analysis, differentially expressed probes with *p* ≤ 0.01 were analyzed for KEGG pathway and Gene Ontology enrichment using DAVID (Huang da et al., 2009).

The data discussed in this publication have been deposited in NCBI’s Gene Expression Omnibus and are accessible through GEO series accession number GSE120548.

## Results

### Effects of Maternal HP/LC and LP/HC Diets on Birth Weights, Perinatal Weights, and Adult Growth Curves

**FIGURE 1:**
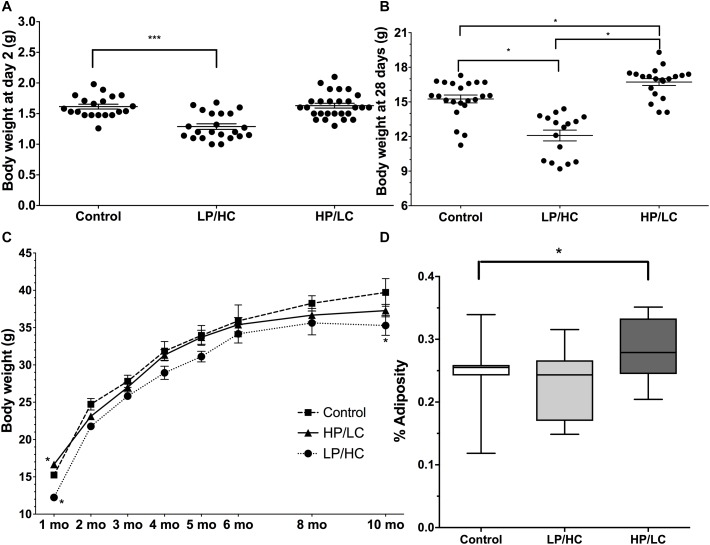
Birth weights, perinatal weights, and growth rates of pups exposed to different *in utero*/perinatal conditions. **(A)** Significantly lower birth weights resulted from *in utero*/perinatal exposure to LP/HC maternal diet (^∗∗∗^ indicates *p* < 0.0001 by Welch’s *t*-test). HP/LC and vehicle were not different from control. Pups were weighed at postnatal day 2. **(B)** Body weights at weaning (postnatal day 28) for male LP/HC and HP/LC, versus control (^∗^ indicates *p* < 0.001 by Welch’s *t*-test). **(C)** Male control, LP/HC and HP/LC, adult growth curves from 1 to 10 months of age. **(D)** Adiposity at 5 months of age showing increased adiposity in HP/LC males (^∗^ indicates *p* < 0.05 by Welch’s *t*-test). Sample sizes range from 15 to 27, 15 to 22, and 9 to 20 for HP/LC, LP/HC, and Control groups, respectively.

*In utero* LP/HC exposure resulted in IUGR as reflected by significantly lower birth weights, whereas HP/LC litter birth weights were not different from control mice (Figure 1A). At weaning, however, HP/LC offspring weighed significantly more than controls (Figure 1B), whereas IUGR LP/HC pups still weighed less at weaning than controls (Figure 1B). IUGR LP/HC weighed significantly less than controls throughout the study, but the higher body weights of HP/LC at weaning did not persist over time and after 3 months HP/LC body weights normalized and were not different from controls (Figure 1C).

### Effects of Maternal HP/LC and LP/HC Diets on Adiposity in Adulthood

In order to compare the impact of *in utero/*postnatal exposure on adult fat storage, we measured adiposity using NMR in the offspring at 5 months of age. HP/LC male offspring had significantly increased adiposity (Figure 1D), which supports previous findings (Thone-Reineke et al., 2006), while LP/HC offspring showed no difference from controls in adiposity (Figure 1D), also supporting previous findings (Daenzer et al., 2002).

### Effects of Maternal HP/LC and LP/HC Diets on Glucose Tolerance and Insulin Response

A key metabolic syndrome indicator is glucose intolerance, which is a measure of baseline glucose, baseline insulin, and the ability to respond to an exogenous glucose load with adequate insulin production. We therefore carried out an Intraperitoneal Glucose Tolerance Test (IPGTT) at 9 months of age.

**FIGURE 2:**
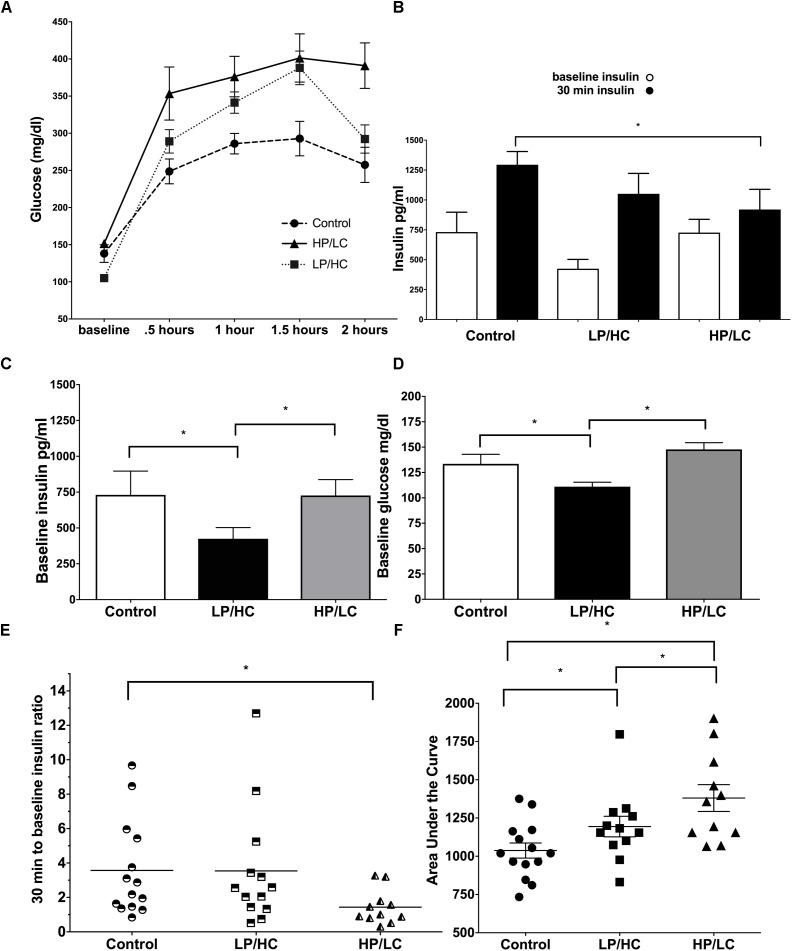
Intraperitoneal Glucose Tolerance Test, baseline glucose, baseline insulin, and insulin response at 9 months of age for males LP/HC and HP/LC vs. control. **(A)** Intraperitoneal Glucose Tolerance Test. **(B)** Average baseline and 30 min insulin levels Baseline Glucose. **(C)** Baseline Insulin. **(D)** Baseline Glucose. **(E)** Individual IRR. **(F)** AUC. Median ratio is indicated by the bar in each treatment group. Sample size ranges are 11–12, 12–13, and 11–15 for HP/LC, LP/HC, and Control groups, respectively. (^∗^ indicates *p* < 0.05 by Welch’s *t*-test).

Early life exposure to LP/HC and HP/LC impaired the ability of offspring to clear glucose in adulthood. Both LP/HC and HP/LC mice had a significantly higher area under the curve (AUC) for blood glucose levels measured at 0.5, 1.0, and 1.5 h post glucose administration (Figure 2A). For LP/HC offspring, both baseline glucose and insulin concentrations were reduced when compared to control (Figures 2B,C), the latter of which likely contributed to the higher AUC for blood glucose (Figure 2D). In contrast, baseline glucose and insulin concentrations of HP/LC offspring were similar to controls; however, at 30 min post glucose injection, the insulin concentrations were significantly lower (Figure 2E), indicating an inability to mount an effective insulin response to the glucose challenge, and resulting in the higher AUC. We also examined the insulin response per individual offspring by using an insulin response ratio (IRR), which was the insulin concentration value at 30 min post glucose challenge divided by the baseline value (Figure 2F). This comparison provided further evidence that the higher AUC in HP/LC male offspring was likely due to an insufficient insulin response, with half of HP/LC animals having an IRR < 1 (lower insulin after the challenge than at baseline) (Figure 2F). Overall, the median IRR was 2.2 for control, 2.3 for LP/HC, and 1.0 for HP/LC (Figure 2F).

### Gene Expression and Pathway Alterations in the Liver and Hypothalamus

The metabolic phenotypic differences in adult offspring were accompanied by significant differences in gene expression in two metabolically relevant tissues, the liver and hypothalamus collected at final dissection conducted at 10 months of age. For the hypothalamus, we identified 1,441 and 843 differentially expressed genes (DEGs) for HP/LC and LP/HC groups, respectively, by Student’s *t*-test using a *p* < 0.01 as the cutoff. For liver, 2014 and 926 DEGs were identified for HP/LC and LP/HC groups, respectively. At a false discovery rate (FDR) < 5%, very few DEGs were identified (2, 2, 14, and 2 genes altered in the LP/HC hypothalamus, HP/LC hypothalamus, LP/HC liver, and HP/LC liver, respectively; Supplementary Tables S5–S8).

We queried the DEGs reaching *p* < 0.01 against canonical pathways curated in KEGG and GO, and found significant over-representation of genes in diverse pathways ranging from metabolic, neural structure, protein modification, to signaling pathways such as MAPK (Table 1). Pathway analysis does not rely on the reliability of individual DEGs but the aggregate activities of multiple DEGs in coherent pathways, thus reducing false positive detection. Compared to the LP/HC group, the HP/LC group had more significant pathways that were expressed in both liver and hypothalamus at Bonferroni-corrected *p* < 0.05. Although the majority of the pathways found in the LP/HC group only reached uncorrected *p* < 0.05 and are considered suggestive pathways here, many overlapped with those from the HP/LC group.

**Table 1:** KEGG and GO pathway enrichment of differentially expressed genes in the hypothalamus, liver or both under different nutritional exposures in early life.

Pathways	Liver		Hypothalamus	
	HP/LC	LP/HC	HP/LC	LP/HC
Phosphate metabolism	4.10E-08	3.40E-04	1.50E-09^∗^	5.10E-04
Biopolymer modification	–	5.80E-04	1.70E-07^∗^	–
Cellular localization of protein	–	3.00E-04	–	7.00E-05
RNA processing	3.70E-06	7.80E-04	–	7.10E-05
Nucleotide processing	–	–	3.80E-05^∗^	–
Neural restructuring	–	–	7.90E-05^∗^	3.00E-03
Protein transport	–	2.00E-04	8.70E-06^∗^	5.00E-05
Cell cycle	1.00E-05	2.10E-04	3.30E-03	–
Synaptic transmission	–	–	1.00E-05^∗^	–
MAPK signaling pathway	–	–	3.60E-05^∗^	–
Gap junction	–	–	7.70E-05^∗^	–
Apoptosis	8.70E-04	–	5.00E-04	1.50E-03
Purine metabolism	5.50E-06^∗^	–	–	–
Hedgehog signaling pathway	–	4.50E-03	–	5.20E-04
Colorectal cancer	1.70E-05^∗^	–	–	–
Embryonic development	–	3.60E-03	3.90E-03	1.60E-03
Oxidative phosphorylation	–	–	3.80E-03	4.30E-05^∗^
Circadian rhythm related pathways	6.30E-05	2.40E-04	1.20E-03	–
Long-term potentiation	–	–	7.90E-05^∗^	–
Response to DNA damage stimulus	1.80E-06^∗^	–	–	–


*Pathways shown are those that passed Bonferroni-corrected p < 0.05 in at least one group or those that are consistent in at least two groups at uncorrected p < 0.05. All p-values are based on one-sided Fisher’s exact test and Bonferroni correction was carried out on KEGG and GO pathways separately. ^∗^ indicates passing the Bonferroni-corrected p < 0.05.*

In both HP/LC and LP/HC conditions, shared KEGG/GO pathways within the hypothalamus include phosphate metabolism, oxidative phosphorylation, neural restructuring, apoptosis, and protein transport. Within the HP/LC condition, KEGG/GO pathways were specifically enriched for synaptic transmission, nucleotide processing, biopolymer modification, MAPK signaling pathway, circadian rhythm, long-term potentiation, and gap junction signaling in the hypothalamus. Within the LP/HC condition, hedgehog signaling was uniquely enriched (Supplementary Tables S1–S4). At FDR < 0.05, only *Ebf1* was significant for the HP/LC condition within hypothalamic tissue. *Ebf1* encodes Early B Cell Factor 1, a transcription factor regulating peroxisome proliferator-activated receptor gamma. Importantly, it has been linked to childhood obesity and neuronal differentiation (Moreno and Bronner-Fraser, 2005; Jimenez et al., 2007; Comuzzie et al., 2012).

The liver transcriptome pathway analysis showed differential gene expression for RNA processing, phosphate metabolism, cell cycle, and circadian rhythm in both nutritionally modified states. Purine metabolism, apoptosis, response to DNA damage stimulus and DNA repair within the liver were uniquely enriched within the HP/LC diets. Several pathways enriched among the genes altered by the LP/HC diet within the liver include biopolymer modification, cellular protein localization/transport, embryonic development, and hedgehog signaling (Supplementary Tables S1–S4). A notable proportion of genes within the enriched pathways in liver are related to the biological clock, including *Wee1*, *Per2* and *Slc5a6*, each previously reported to be associated with the development of metabolic syndrome (Englund et al., 2009; Bechtold et al., 2010; He et al., 2016). Both *Wee1* and *Per2* had an FDR of <5%, while *Slc5a6* had an FDR of 0.056, all representing top DEGs in liver. Additional top liver DEGs at FDR < 5% are *Ndrg1* and *Rorγ* (Supplementary Tables S5–S8). *Ndrg1* (*N*-Myc Downstream Regulated 1) has been shown to promote the differentiation of adipocytes from precursor cells through the induction of *Pparγ* (peroxisome proliferator-activated receptor) expression (Cai et al., 2017). A single nucleotide polymorphism (SNP) within *Rorγ* (RAR-related orphan receptor gamma) has been implicated in potentially contributing to T2DM susceptibility (Wang et al., 2003). Another study also demonstrated several SNPs within *Rorγ* to be associated with obesity in cattle (Barendse et al., 2006).

Across the dietary groups and the two tissues, consistent pathways include circadian rhythm, phosphate metabolism, RNA processing, cell cycle/apoptosis, and embryonic development.

## Discussion

Our study showed that maternal diets with abnormal nutritional compositions such as HP/LC or a LP/HC diet led to developmental differences, which persisted into adulthood affecting glucose metabolism, body weight, adiposity and gene expression levels in the hypothalamus and liver. Male offspring from mothers exposed to LP/HC diet showed a significantly reduced birth weight, which persisted at weaning (day 28) and up to 10 months of age. Male offspring from the HP/LC group trended toward a higher birth weight, and had a significantly higher body weight at weaning and increased adiposity at 5 months. Additionally, both sets of offspring were found to have an impaired ability to clear glucose in adulthood. The IPGTT conducted in HP/LC and LP/HC litters resulted in a significantly higher AUC for both groups compared with control. Interestingly, the LP/HC diet presented offspring with reduced baseline insulin and glucose levels, whereas the HP/LC diet had a reduced insulin response 30 min after glucose exposure. This led us to investigate what genes and pathways might underlie these phenotypic differences in the offspring. We found diet- and/or tissue-specific genes and pathways (e.g., gap junction in hypothalamus and purine metabolism in liver for HP/LC, neuronal pathways for both diets in hypothalamus, hedgehog signaling for LP/HC in both tissues), as well as shared pathways across tissues and diets (e.g., cell cycle and circadian rhythm).

There is a well-cataloged history of studies exploring the effects of maternal diet alterations on diseases in adult offspring, specifically within the context of metabolic perturbations (Langley-Evans et al., 1994; Rees et al., 2000; Daenzer et al., 2002; Zambrano et al., 2006). Previous reports have associated both maternal high- and low-protein diets with increased blood pressure, while maternal LP diet results in reduction of body weight in offspring (Jahan-Mihan et al., 2015). Our results indicate that the inadequacy of the low protein maternal diet induces low birth weight and lower body weights throughout life but no difference in adiposity. Additionally, consistent with our previous findings, LP/HC offspring had diminished glucose clearance rates (Bhasin et al., 2009). The HP/LC maternal diet outcomes indicated that too much stimulation of the nutrient-sensing pathways *in utero*, while not resulting in higher birth weights, did result in higher body weight at weaning, and higher adiposity and glucose intolerance in adulthood. There are conflicting results regarding the physiological impact of increased maternal protein intake on offspring in previous studies. One study exploring high protein maternal diet on offspring reported no change in body weight, degree of adiposity, or glucose intolerance (Thone-Reineke et al., 2006), while another reported a reduction in body weight with an increase in adiposity (Daenzer et al., 2002). Differences in results may be due to variations in maternal dietary composition, specifically the ratio of protein to complementary nutrients and the total energetic intake; one diet had a protein to carbohydrate ratio of 1.12 while the other was 0.91. Our study utilized a protein-carbohydrate ratio of 0.35, which is significantly lower than the other two studies and may contribute to the variation in results recorded by different groups.

In our study, we essentially found a U-shaped curve in terms of the relationship between maternal protein intake and glucose tolerance in the offspring’s adult life. It is predicted that optimal levels for many essential nutrients follow a U-shaped curve: levels that are too low as well as too high result in detrimental health outcomes. Sufficient vitamin A, for instance, is essential in pregnancy to prevent diaphragmatic hernia in the fetus (Clugston et al., 2010), but excess vitamin A results in cleft palate (Ackermans et al., 2011). High protein diets have been advocated as a way to achieve weight loss or prevent weight gain in humans, and our findings suggest that this type of diet, while effective in preventing weight gain, would not be beneficial to the developing offspring. Furthermore, the HP/LC diet resulted in high baseline glucose levels compared with controls at 10 months of age. Therefore, the current trend of providing high protein to low birth weight infants in Neonatal Intensive Care Units (Hay and Thureen, 2010) to combat postnatal growth restriction, may have far reaching implications to these infants that may prove detrimental toward subsequently developing glucose intolerance, and needs close monitoring upon follow-up.

Our hypothalamic transcriptome and pathway analyses in the offspring indicate alterations in tissue gene programming, likely through the developmental consequences resulting from embryonic nutritional imbalances, that connects maternal diet with long-term physiological outcomes in offspring. Key hypothalamic pathways such as synaptic transmission, neural restructuring and neural development were particularly altered by the HP/LC maternal diet suggesting that these pathways may lead to abnormal neural circuitry in the hypothalamus, which plays a key role in energy balance, ultimately contributing to dysregulation of metabolic homeostasis including increased fat storage and glucose intolerance in adulthood. These findings corroborate a recent study where a high fat maternal diet led to significant alterations in hypothalamic gene expression, resulting in a disruption of regular hypothalamic circuitry through ECM and dopamine availability pathway perturbations (Barrand et al., 2017). While the pathways are not the same between maternal diets, they all reflect changes in neuronal signaling due to maternal dietary alterations. In addition, mitochondrial dysfunction, specifically with regard to oxidative phosphorylation within the hypothalamus has been reported to be associated with neurodegeneration and anorexia in anx/anx mice (Lindfors et al., 2011). This seems to link with our data showing enrichment of oxidative phosphorylation pathways within hypothalamic tissue for both diets and the development of feeding aberrations observed in a previous study (Lindfors et al., 2011). Finally, our finding that *Ebf1* expression is significantly altered by HP/LC maternal diet is suggestive of a metabolic disorder and dysregulation of neural development, as previous studies have linked this gene with childhood obesity and neuronal differentiation (Moreno and Bronner-Fraser, 2005; Comuzzie et al., 2012).

In the liver, circadian rhythm and metabolic pathways such as phosphate metabolism and metabolic regulation were prominent, indicating that these pathways may be major effectors of metabolic syndrome outcomes due to altered maternal diets. Our findings that a number of circadian rhythm related pathways (Table 1), including rhythmic processes and transcriptional regulation via *Per2*, are altered within hepatic tissue under a LP/HC maternal diet is an interesting outcome of the current study and is supported by previous work done in intrauterine growth restricted male rat models (Freije et al., 2015). Additionally, physiological evidence directly supports the role of circadian rhythm dysregulation in increasing the risk for obesity, type 2 diabetes, and other metabolic syndrome traits (Blattler et al., 2012), thus supporting our physiological findings. Notably, around 10% of transcripts oscillate diurnally within the liver (Panda et al., 2002), many of which are involved in energy homeostasis and metabolism, further supporting the potential pathophysiological repercussions of hepatic circadian dysregulation. Furthermore, the expression of *Slc5a6*, a biotin transporter, is dependent on cell autonomous circadian clock genes *Clock* and *Bmal1* (He et al., 2016). Notably, biotinylation is an essential posttranslational modification of a number of mammalian carboxylases, including acetyl-CoA carboxylase, which has been linked to non-alcoholic fatty liver disease (Tessari et al., 2009).

We also report the alteration of MAPK (for HP/LC) and hedgehog (for LP/HC) signaling pathways, both of which have been previously linked with the facilitation of insulin signaling. Disruption of insulin production or transduction of its signal is intimately associated with generation of metabolic syndrome (Guo, 2013). More specifically, our data suggests that the HP/LC maternal diet disrupts the MAPK activity within the hypothalamus. MAPK facilitates hypothalamic insulin signaling involved in determining the organism’s energy and nutrient homeostasis (Mayer and Belsham, 2009; Schultze et al., 2012). The hedgehog-signaling pathway was predominantly affected by the LP/HC condition for both the liver and hypothalamus. The activation of this pathway is highly correlated with the severity of liver damage in patients with non-alcoholic fatty liver disease and is closely linked with hepatic repair mechanisms. Additionally, hedgehog perturbation is involved in both congenital and adult onset metabolic syndrome (King et al., 2008; Choi et al., 2011; Guy et al., 2012).

We acknowledge that this is a discovery study and the findings provide the framework for future avenues of investigation. In particular, pathohistological and structural analysis of both the hypothalamus and liver tissues are necessary to show association between the highlighted pathways/genes and tissue pathology. Additionally, protein level assays and perturbation experiments are necessary to validate the functional and physiological outcomes of the potential causal genes highlighted by our current study. Moreover, it would be interesting to explore further the classification of adiposity and the relevant abundance of white versus brown adipose tissue. This would help characterize more precisely the effects of nutritional perturbation on adipogenesis. A potential limitation of this study is a lack of further validation of our Affymetrix data via RT-PCR, although the validity of microarrays has been highly supported in the literature (Morey et al., 2006; Wang et al., 2006). Additionally, our study mainly focused on identifying the pathways perturbed by diets instead of individual DEGs. The reliability for the significant pathways is high because the random chance to have multiple significant DEGs from the same pathway is extremely low.

Overall, the prevalence of metabolic disorders such as T2DM and metabolic syndrome in modern societies is growing and the etiology is clearly complex, involving diet, physical activity, and possibly endocrine disrupting chemicals in the environment (De Coster and van Larebeke, 2012). Here, we demonstrate that either a deficiency or an excess of protein in early life can result in adverse effects on metabolic outcomes in adulthood, especially with respect to glucose intolerance and T2DM-like phenotypes. Importantly, these early life perturbations altered gene pathways in adulthood, which may underlie the observed metabolic dysregulation, although the cause and effect relationship remains to be further explored. Our unique study design directly comparing the effects between low and high protein maternal intake on both metabolic phenotypes of the offspring and multi-tissue transcriptomes offers a comprehensive understanding of the role of maternal protein imbalance in metabolic health. Based on our findings, a balanced approach to maternal and postnatal nutrition toward achieving optimal outcomes is necessary for a normal physiological phenotype in the offspring.

## Author Contributions

LJM designed and conducted the experiments, researched the data, analyzed all the data, and drafted the manuscript. QM, SL, and CP analyzed the gene expression data. MB analyzed and summarized all the data, and wrote the manuscript. SX and WT conducted the experiments. JW researched and analyzed the data. SD developed experimental design and assisted in phenotypic experiments and interpretation. AJL developed the experimental design. XY supervised the data analysis and manuscript writing. All authors reviewed and edited the manuscript.

## Conflict of Interest Statement

The authors declare that the research was conducted in the absence of any commercial or financial relationships that could be construed as a potential conflict of interest.
